# Making sense of diabetes medication decisions: a mixed methods cluster randomized trial using a conversation aid intervention

**DOI:** 10.1007/s12020-021-02861-4

**Published:** 2021-09-09

**Authors:** Marleen Kunneman, Megan E. Branda, Jennifer L. Ridgeway, Kristina Tiedje, Carl R. May, Mark Linzer, Jonathan Inselman, Angela L. H. Buffington, Jordan Coffey, Deborah Boehm, James Deming, Sara Dick, Holly van Houten, Annie LeBlanc, Juliette Liesinger, Janet Lima, Joanne Nordeen, Laurie Pencille, Sara Poplau, Steven Reed, Anna Vannelli, Kathleen J. Yost, Jeanette Y. Ziegenfuss, Steven A. Smith, Victor M. Montori, Nilay D. Shah

**Affiliations:** 1 Knowledge and Evaluation Research Unit, Division of Endocrinology, Diabetes, Metabolism, and Nutrition, Mayo Clinic, Rochester, MN USA; 2 Medical Decision Making, Department of Biomedical Data Sciences, Leiden University Medical Center, Leiden, The Netherlands; 3 Department of Biostatistics and Informatics, Colorado School of Public Health, University of Colorado-Denver Anschutz Medical Campus, Aurora, CO USA; 4 Division of Biomedical Statistics and Informatics, Department of Health Sciences Research, Mayo Clinic, Rochester, MN USA; 5 Division of Health Care Policy and Research, Department of Health Sciences Research, Mayo Clinic, Rochester, MN USA; 6 Laboratoire d’anthropologie des enjeux contemporains, Lyon, France; 7 Faculty of Public Health and Policy, London School of Hygiene and Tropical Medicine, London, UK; 8 Department of Medicine, Hennepin Healthcare and University of Minnesota, Minneapolis, MN USA; 9 Mayo Clinic Robert D. and Patricia E. Kern Center for the Science of Health Care Delivery, Rochester, MN USA; 10 Department of Psychiatry and Psychology, Mayo Clinic Health System, Mankato, MN USA; 11 Department of Family Medicine and Community Health, University of Minnesota Medical School, Minneapolis, MN USA; 12 Practice-Based Research Network, Mayo Clinic, Rochester, MN US; 13 Center for Translational Science Activities, Mayo Clinic, Rochester, MN USA; 14 Center for Patient and Provider Experience, Hennepin County Medical Center, Minneapolis, MN USA; 15 School of Nursing, University of Minnesota, Minneapolis, MN USA; 16 Decision Partners for Health, Richfield, MN USA; 17 Mayo Clinic Health System Northwest Wisconsin, (dept) Home Health and Hospice, Eau Claire, WI USA; 18 Department of Family and Emergency Medicine, Faculty of Medicine, Laval University, Quebec, QC Canada; 19 Park Nicollet International Diabetes Center, St. Louis Park, MN USA; 20 Mayo Clinic Health System, La Crosse, WI US; 21 Kern Center for the Science of Health Care Deliver, Mayo Clinic, Rochester, MN USA; 22 Office of Professional Worklife, Hennepin Healthcare, Minneapolis, MN USA; 23 Department of Internal Medicine, Park Nicollet Clinic, Brooklyn Center, MN USA; 24 Division of Epidemiology, Department of Health Sciences Research, Mayo Clinic, Rochester, MN USA; 25 Division of Health Sciences Research, Mayo Clinic, Rochester, MN USA; 26 Center for Evaluation and Survey Research, HealthPartners Institute, Bloomington, USA; 27 Division of Endocrinology, Diabetes, Metabolism, and Nutrition, Mayo Clinic, Rochester, MN USA

**Keywords:** Shared decision making, Decision aids, Patient–clinician communication, Diabetes, Patient-centered care

## Abstract

**Purpose:**

To determine the effectiveness of a shared decision-making (SDM) tool versus guideline-informed usual care in translating evidence into primary care, and to explore how use of the tool changed patient perspectives about diabetes medication decision making.

**Methods:**

In this mixed methods multicenter cluster randomized trial, we included patients with type 2 diabetes mellitus and their primary care clinicians. We compared usual care with or without a within-encounter SDM conversation aid. We assessed participant-reported decisions made and quality of SDM (knowledge, satisfaction, and decisional conflict), clinical outcomes, adherence, and observer-based patient involvement in decision-making (OPTION12-scale). We used semi-structured interviews with patients to understand their perspectives.

**Results:**

We enrolled 350 patients and 99 clinicians from 20 practices and interviewed 26 patients. Use of the conversation aid increased post-encounter patient knowledge (correct answers, 52% vs. 45%, *p* = 0.02) and clinician involvement of patients (Mean between-arm difference in OPTION12, 7.3 (95% CI 3, 12); *p* = 0.003). There were no between-arm differences in treatment choice, patient or clinician satisfaction, encounter length, medication adherence, or glycemic control. Qualitative analyses highlighted differences in how clinicians involved patients in decision making, with intervention patients noting how clinicians guided them through conversations using factors important to them.

**Conclusions:**

Using an SDM conversation aid improved patient knowledge and involvement in SDM without impacting treatment choice, encounter length, medication adherence or improved diabetes control in patients with type 2 diabetes. Future interventions may need to focus specifically on patients with signs of poor treatment fit.

**Clinical trial registration:**

ClinicalTrial.gov: NCT01502891.

## Background

Over the last few decades, there has been an increasing call for care to be more patient-centered and for patients to be more involved in their care [1, 2]. One way of achieving this is for patients and clinicians to engage in shared decision making (SDM) [3]. In SDM, patients and clinicians work together to understand the patient’s situation and determine how best to address it [4].

How to review and use evidence to shape the clinical response to the patient’s situation remains a key challenge in the implementation of SDM in practice [5]. Evidence suggests that SDM tools designed for use within the clinical encounter by patients and clinicians—sometimes called conversation aids—can support sharing evidence, improving patient knowledge, and facilitating SDM [6]. When properly designed, these tools only contain the information necessary, ideally from a systematic and up-to-date evidence summary, to support the patient–clinician conversation [7, 8]. Clinicians and patients, working together, can draw from this information to co-create a treatment plan that makes sense and responds well to the patient’s situation.

We previously developed a within-encounter SDM tool, the Diabetes Medication Choice, to help patients with type 2 diabetes and their clinicians decide about diabetes medication [9]. In two pilot randomized trials vs. usual care, the use of this tool improved patient knowledge, patient involvement in decision making, and patient comfort with the decision made [10, 11]. However, these trials did not show improvement in glycemic control. This may have been due to a lack of effectiveness of SDM, or to an inability to find that effect either because these trials were not powered to assess these outcomes or because the population selected had limited chance to improve given that they were relatively healthy, highly educated, and demonstrated high treatment adherence [10, 11]. Possibly, larger trials enrolling more diverse populations may provide a better opportunity to assess the downstream effects of support SDM and more generalizable evidence of the impact of conversation aids such as the Diabetes Medication Choice in translating evidence into patient-centered care and outcomes.

The aim of this study was to determine the effectiveness of the Diabetes Medication Choice SDM conversation aid versus guideline-informed usual care in translating evidence about diabetes medications into routine primary care. Also, in order to better understand personal factors that might underpin SDM conversations, we explored patients’ perspectives about medication decision making in the context of diabetes management.

## Methods

### Study design

We conducted a practical mixed methods cluster (practice) randomized trial, comparing the use of the Diabetes Medication Choice conversation aid (intervention) to guideline-informed usual care in primary care practices among patients with poorly controlled type 2 diabetes (ClinicalTrial.gov: NCT01502891). The Mayo Clinic Institutional Review Board, along with the boards of participating sites, approved the study (#10-006952).

#### Randomization

We paired practices according to their numbers of clinicians (≤2 vs. >2) and prevalence of diabetes in their practice (self-rated as high vs. low). The study statistician randomly allocated practices to the intervention or usual care after practices had been enrolled, ensuring allocation concealment. Practices, clinicians, and investigators were aware of the assigned arms.

#### Intervention and usual care

The intervention consisted of the use of the Diabetes Medication Choice conversation aid by patients and clinicians during the clinical encounter. This tool presents general considerations and adverse effects of diabetes medication, organized in terms by topics that matter to patients: weight change, daily routine, blood sugar levels (HbA1c), daily blood sugar testing, hypoglycemia, and cost. The latest version of the tool is freely available at https://diabetesdecisionaid.mayoclinic.org/. Each topic occupies a card that the patient and clinician review as needed, with both negotiating which ones to review, in what order, and in what detail, until they arrive at a preferred approach by consensus. Patients could take home a one-page handout version of the conversation aid. Clinicians received training on how to use the conversation aid during a 10 min group session (including rationale, demonstration of use, role playing), by accessing an online demonstration and a one-page storyboard, and by requesting ad-hoc, one-on-one training during the study.

Usual care consisted of clinicians engaging with their patients as usual with an increased awareness of diabetes care guidelines. To that end, we provided clinicians with copies of the Institute for Clinical Systems Improvement diabetes care algorithm, a set of guidelines promoted by health plans in Minnesota, and the American Diabetes Association-International Diabetes Federation expert algorithm to assist in in choosing an antihyperglycemic agent [12]. These informational resources were also provided to clinicians in the intervention group.

### Participants and setting

This study took place between July 2010 and May 2014 across 20 rural, suburban, and inner-city primary care practices from six health systems in the Midwest (Minnesota, Wisconsin), United States. All clinicians in participating practices caring for patients with type 2 diabetes were eligible to participate. Patients were eligible if they were an adult with a clinical diagnosis of type 2 diabetes mellitus, a recent (<12 months) HbA1c measure greater than 7.3% (selected by consensus to represent a level above the recommended 7% target HbA1c at the time of the trial), not receiving insulin (to indicate that they had several treatment options available to improve glycemic control, including insulin), a pre-scheduled appointment with a primary care clinician participating in the study, no intellectual or sensorial barriers to providing written informed consent, and available for the duration of follow-up (12 months).

### Procedure

We identified potential practices from public listings of regional practices and approached either the medical director or a pre-identified clinical champion to seek interest in participation. If interested, we presented the study at their on-site staff meeting. If the practice agreed to participate, we immediately recruited clinicians at this meeting and, in addition, individually approached clinicians. On-site study coordinators then identified potential eligible patients and contacted them either via phone or in-person immediately preceding their scheduled appointment to gauge interest in participating. All participants provided written informed consent.

We collected participant demographics at time of consent. With permission, we video-recorded patient–clinician encounters. We asked patients and clinicians to complete a paper survey immediately following the encounter, and we asked patients to complete follow-up paper surveys 2, 6, and 12 months after the encounters. Lastly, we reviewed patients’ medical records at baseline, and at 12 months after the encounter to collect information about hemoglobin A1c and use of diabetes medications. Patients were also asked to provide consent for the research team to contact the pharmacy where the patients usually fill their medication prescriptions to document dates of medication fills.

At baseline, we invited and consented patients to participate in an interview, which took place at a later date either in-person in the clinic or by telephone, based on the patient’s preference. Interviewers contacted consenting patients for scheduling, aiming to balance the interviews by study arm. We aimed to conduct interviews as soon after the clinic visit as possible, to promote more accurate recall. Interviews were recorded with permission, transcribed verbatim, and checked against audio files for accuracy.

### Measures

#### Decision and quality of decision-making

In the post-encounter survey, patients reported whether a discussion about diabetes treatment occurred and, if so, what decision was made (e.g., increase medication dose, change, or add a medication). They also completed six true/false knowledge items about the treatment options and their pros and cons [10, 11, 13], our primary SDM outcome, three of the five subscales of the Decisional Conflict Scale (perceived knowledge, support, and effectiveness) [14], and two items on satisfaction with the information shared [11].

Clinicians’ post-encounter survey consisted of one item on satisfaction with decision making [10, 11]. Clinicians in the intervention arm also answered two questions about the ease of incorporation the conversation aid into the encounter.

From the recordings of encounters, we assessed the extent to which clinicians engaged patients in the decision-making process, using the 12-item Observing Patient Involvement in Decision-Making (OPTION) scale [15]. We also assessed the extent to which clinicians covered the topics described in the conversation aid in both intervention and control arm, using an 11-item fidelity checklist. We considered adequate fidelity when patients and clinicians covered >7 items [11, 16]. Finally, we noted the length of the encounter.

#### Clinical and adherence outcomes

Our primary clinical outcome was the proportion of patients having reached a level of hemoglobin A1c (HbA1c) of <7.3%. We used the HbA1c measure that had been taken at the closest point in time to the final follow-up visit (12 months from enrollment) that was available in the medical record. We calculated, from pharmacy records, primary adherence as the proportion of patients who filled their prescription of antihyperglycemic medications within 30 days of the index encounter, and secondary adherence (i.e., persistence) as both the average of percentage of days covered (PDC) and the proportion of patients with a PDC >80% [17]. PDC was defined as the number of days a patient had a supply of each medication divided by the number of days of eligibility for that medication, for each antihyperglycemic medication prescribed [17].

#### Patient interview

Two members of the study team (JL and KT) completed the patient interviews using a semi-structured interview guide developed using Normalization Process Theory (NPT) as a guiding framework [18]. NPT focuses on the implementation of complex interventions in healthcare. For this study, NPT constructs informed questions about the work patients do to manage their health and make decisions about care [19]. The goal of this qualitative part of the study was to shed light on patient chronic disease management, which includes interactions with the healthcare team for patients in both study arms. We asked patients in the intervention arm additional questions about satisfaction with the conversation aid and its role in decision making. For in-person interviews with patients whose clinical encounter was recorded, the interviewer played a short clip of the encounter. The interview guide is included as Appendix A.

### Sample size

We sought to estimate the effect of the intervention on patient knowledge, HbA1c, and adherence. We expected that 40% of patients in the usual care arm would reach a HbA1c ≤ 7.3 and established through consensus that, for the intervention to be considered a success, we would require at least 55% of patients in the intervention arm to have reached this target [20]. In order to detect an increase of 15% in the intervention arm at a significance level of 0.05 and 80% power with a two-sided *t* test and a correlation of outcomes [intracluster correlation coefficient (ICC)] within practices of 0.025 (estimated from the DAD (decision aids to enhance SDM for diabetes) trial using the same intervention [11]), we estimated that we would need 660 patients (30 patients per practice, 22 practices total) [21]. Under similar assumptions, we calculated that this sample size had a 99% power to detect one standard deviation difference in any continuous measure (i.e., knowledge), and 80% power to detect a 30% difference in 12-month adherence rates (assuming a control adherence rate of 50%).

For the qualitative part of the study, we aimed to include two patients per participating practice. Interview sampling and data collection occurred iteratively with analysis. The study team met regularly to discuss emergent findings and assess the need for additional data. We stopped the sampling and interviews when the study team found no new insights emerging from subsequent interviews (data saturation).

### Analysis

#### Statistical analysis

The study adhered to the intention-to-treat principle, with patients and clinicians kept and analyzed in the arm to which they were randomized regardless of the intervention received. Patients without an assessment of their HbA1c during the 12-month window following the index encounter were considered to have not met HbA1c <7.3%; patients without adherence data were considered to have not met the 80% PDC cutoff. For all other outcomes, only complete data were analyzed. Sensitivity analyses and adherence were also only conducted on complete data. All analyses accounted for clustering (of practice) in the study design. Patient and clinician characteristics were compared between randomization arms using the cluster-adjusted *t* tests and chi-square tests.

For unadjusted comparisons of outcomes, we used cluster-adjusted *t* tests and chi-square tests, and for all adjusted analyses we used hierarchical generalized linear models [21]. These statistical methods address the unit of analysis issue through terms for each level of grouping or clustering. Also, these models account appropriately for clustering of patients within clinicians and practices and allow us to deal with repeated observations of patients. Analysis was conducted using SAS software 9.4 and Stata 14.0 [22, 23].

#### Qualitative analysis

Three investigators of the study team (JL, JR, and KT) analyzed transcripts of interview recordings using a framework approach. First, investigators familiarized themselves with the data and began to identify key issues and conceptualize a thematic framework, which included a priori topics from the interview guide. The analysis team identified talk about how patients perceive their ability to engage in SDM as an important theme, and they refined the framework to include patient competencies reported in literature about *informed* shared decisions (e.g., capacity to define and establish relationships with clinicians, articulate problems, access and evaluate information, and negotiate decisions with clinicians) [24]. The framework was systematically applied to transcripts by at least two investigators, with discrepancies discussed until consensus was reached.

Transcripts were entered into NVivo 12.0 to facilitate queries and analysis, which involved charting the data thematically by study arm (using a coding matrix) and then returning to the study questions during interpretation. During this stage the investigators also checked for differences related to conversation aid use.

## Results

### Participants

A total of 350 patients from 20 practices were enrolled from February 2011 through June 2013. Enrollment ended before meeting the recruitment goal due to slower than anticipated accrual.

**Fig. 1:**
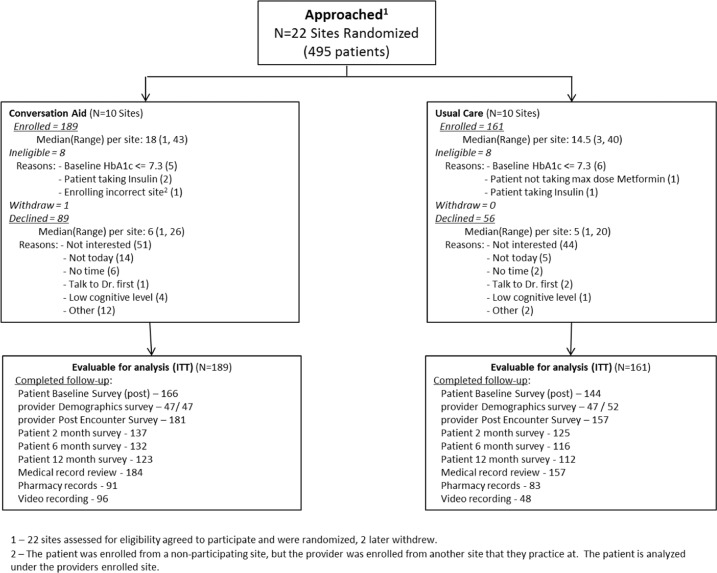
Study flowchart

Twenty-two sites were invited and agreed to participate. Two sites withdrew after being randomized (one in each study arm), due to changes in staffing within each practice. A total of 495 patients were initially assessed as eligible and were approached for study participation (Fig. 1). Of these, 350 (71%) participated, 189 in the intervention arm, and 161 in the usual care arm. A total of 99 clinicians participated in the trial, four of them seeing patients at two sites for a final total of 103 clinicians. All patient and clinician characteristics were balanced across arms (Table 1). Appendix B reports the results of the 2- and 6-month patient surveys (on reuse of the Diabetes Medication cards, recall, and adherence to the decision and to the medicines).

**Table 1:** Participant characteristics

Patient characteristics	Usual care (*N* = 161)	Conversation aid (*N* = 189)
Average per site	16	17
Age, mean (SD)	62 (12)	59 (11)
Female, *N* (%)	86 (53)	72 (38)
BMI, mean (SD)^a^	36 (7.3)	36 (8.9)
Race, *N* (%)		
White	139 (86)	155 (82)
Black	14 (9)	24 (13)
Other	8 (5)	10 (5)
Insurance, *N* (%)^b^		
Employer/Union	69 (49)	73 (47)
Direct from insurance company	16 (11)	21 (14)
Medicare	30 (21)	33 (21)
Medicaid	13 (9)	23 (15)
Tricare/military	2 (1)	0 (0)
Other	10 (7)	4 (2)
Education, *N* (%)^b^		
High school or less	50 (35)	66 (42)
Vocational/4-year college degree	84 (59)	76 (49)
Graduate degree	8 (6)	14 (9)
Adequate literacy, N(%)^b^	24 (17)	21 (13)
HbA1c, mean (SD)	8.9 (1.2)	8.9 (1.4)
HbA1c, *N* (%)		
<7.5%	14 (9)	11 (6)
7.5 to <8.0%	34 (21)	37 (20)
8.0 to 8.5%	25 (16)	48 (25)
>8.5%	88 (55)	93 (49)
Years with diabetes, *N* (%)^b^		
<5	38 (27)	49 (31)
5 to <10	53 (38)	58 (36)
>10	49 (35)	53 (33)
Years with clinician, *N* (%)^b^		
<1	31 (22)	30 (18)
1 to <5	33 (24)	51 (31)
5 to <10	23 (16)	41 (25)
>10	53 (38)	44 (27)
**Clinician demographics**	**Usual care** **(*N*** = 53)	**Conversation aid** **(*N*** = 48)
Clinicians per site		
Mean (SD)	5.3 (3.5)	4.8 (3.8)
Median (Q1–Q3)	5.5 (2–7)	4 (3–5)
Age, mean (SD)^c^	45 (12)	45 (11)
Female, *N* (%)	27 (51)	26 (54)
Practice, *N* (%)		
Family medicine	44 (83)	36 (75)
Internal medicine	9 (17)	11 (23)
Other	0 (0)	1 (2)
Clinician Type^d^, *N* (%)		
Physician (MD, OD)	46 (87)	39 (77)
Advance practitioner (PA/NP)	6 (11)	7 (15)
Years in practice, mean (SD)^d^	12 (12)	12 (910)
Patients in panel with diabetes, *N* (%)^e^		
<25% of the panel	30 (57)	29 (60)
25–50%	15 (28)	12 (25)
>50%	5 (9)	2 (4)
Number of encounters, mean (SD)	3 (3)	4 (3)

^a^Usual care *N* = 144, Conversation aid *N* = 170

^b^Self-reported by patients, missing responses are not represented in counts or percentages.

^c^Usual care: *N* = 51, Conversation aid: *N* = 43

^d^Usual care: *N* = 52, Conversation aid: *N* = 44

^e^Usual care: *N* = 50, Conversation aid: *N* = 43

We completed qualitative interviews with 26 patients (11 in usual care arm, 15 in intervention arm). Patients’ ages ranged from 35 to 73 years (mean 59), and ten (39%) were female. The average length of the interviews was 27 min (range 12–44 min), the median time between the index encounter and the interview was 42 days (range 9–137 days).

**Table 2:** Participant-reported and observed encounter outcomes

Outcomes (Usual care N/Conversation aid N)	Usual care	Conversation aid	Mean difference DA-UC (95% CI)	ICC	*P* value
**Decision made**	***N*** **(%)**	***N*** **(%)**		0.107	0.95
Start new/continue medication	22 (14)	37 (19.6)			
Start new medication only	42 (26)	39 (21.6)			
Continue medication only	88 (55)	98 (51.9)			
Not start new (medication naïve)	1 (1)	2 (1.1)			
No decision made/unsure	8 (5)	13 (6.9)			
**Quality of decision making**	**Mean (95% CI)**	**Mean (95% CI)**			
Knowledge (141/166), % correct	45 (42, 49)	52 (49, 55)	6.2 (0.004, 12.0)	0.025	**0.04**
DCS informed (134/158)	19 (16, 22)	15 (12, 17)	−4.4 (−9.6, 0.9)	0.033	0.10
DCS support (134/157)	15 (13, 18)	15 (13, 18)	−0.2 (−4.3, 3.8)	0.005	0.90
DCS effective (130/152)	15 (12, 18)	15 (12, 17)	−0.01 (−5.7, 5.1)	0.051	0.91
Patient satisfaction, *N* (%)					
*With information re medication* (128/151)	113 (88)	142 (94)	~	0.022	0.15
* With conversation* (129/152)	124 (96)	146 (96)	~	0	0.98
**Video-analysis outcomes**	**Mean (95% CI)**	**Mean (95% CI)**			
OPTION12 (48/96)	17 (15, 20)	25 (23, 27)	7.3 (2.9, 11.8)	0.063	**0.003**
Covered >7 of 11 items (48/96)	42 (35, 49)	68 (63, 73)	25.8 (14.5, 37.2)	0.065	**<0.001**
Encounter duration, min (137/172)	28 (20, 37)	26 (16, 35)	−2.7 (−14.5, 9.1)	0.326	0.64
**Glycemic control**					
HbA1c at 12 months, mean (SD)^a^	8.4 (1.7)	8.1 (1.5)	0.33 (−0.10, 0.76)	0.009	0.13
≤7.3%, intention-to-treat, *N* (%)^b^	33 (20)	50 (26)	~	0.033	0.32
≤7.3%, complete data only, *N* (%)	33 (26)	50 (33)	~	0.058	0.43
**Adherence to medications**					
Mean PDC (SD)^c^	89% (19)	94% (12)	5.0% (−2.0, 12.0)	0.064	0.15
PDC > 80%, intention-to-treat, *N* (%)^b^	67 (42)	80 (42)	~	0.124	0.95
PDC > 80%, complete data only, *N* (%)	67 (81)	80 (87)	~	0.116	0.49
by baseline HbA1c, *N* (%)					
<7.5%	6 (86)	2 (50)	~	0.446	0.26
7.5–7.9%	10 (83)	11 (85)	~	0.377	0.95
8.0–8.5%	12 (86)	20 (91)	~	0.175	0.69
>8.5%	39 (78)	47 (89)	~	0.152	0.36
**Clinician-reported outcomes**	**Usual care** **(*N*** = 155**)**	**Conversation aid** **(*N*** = 176**)**			***P*** **value**
Satisfied with discussion, *N* (%)	133 (86)	155 (88)			0.14
Used conversation aid, *N* (%)^d^	0 (0)	151 (86)			~
Aid was (very) easy to use, *N* (%)^e^		130 (86)			~
Aid was (very) easy to integrate in work with coworkers, *N* (%)		95 (63)			~

*DCS* Decisional Conflict Scale, *SD* Standard Deviation, *PDC* Percentage of Days Covered

^a^Analysis of complete data only

^b^Intent to treat analysis where missing observations were treated as >7.3 or adherence ≤80%

^c^Includes all patients for whom a pharmacy record was received (*N* = 175; UC = 83)

^d^Missing nine responses in Usual care, 1 in Conversation aid

^e^Missing 12 responses

Bold values indicate significance at the 0.05 level

### Decision and quality of decision-making (Table 2)

Clinicians allocated to the intervention arm reported using the conversation aid in 86% of the associated encounters. Of the list of 11 topics expected to be covered as part of an SDM conversation, 68% of recorded intervention encounters and 42% of usual care ones completed >7. Nearly all patients in both arms made a decision to either continue their current medications or to add a new medication, with only 4.9% in the usual care arm and 6.9% in the intervention arm remaining unsure or unable to make a decision. Use of the conversation aid did not significantly alter treatment decisions.

The use of the conversation aid increased post-encounter patient knowledge (correct answers, 52% vs. 45%, *p* *=* 0.02). No significant between-arm differences were found in patients’ perception of being informed (*p* = 0.10) or supported (*p* = 0.90), having made a good decision (*p* = 0.96), or being satisfied with the information given (*p* = 0.15). Use of the conversation aid significantly increased clinicians’ effort to involve patients in decision making (mean difference in OPTION12 scores across arms, 7.3, 95% CI 3, 12; *p* = 0.003). The encounters in both arms were of similar duration (26 min with decision aid vs. 28 min without, *p* = 0.64).

There was no significant difference in clinician satisfaction between arms. Clinicians in the intervention arm reported the conversation aid was (very) easy to use (86% of encounters) and (very) easy for their coworkers to incorporate it into their workflow (63%).

### Clinical outcomes and adherence

Compared to usual care, the use of the conversation aid did not improve glycemic control (HbA1c ≤ 7.3 usual care 20% vs. 26% conversation aid; *p* = 0.32) or medication adherence (PDC 89% vs. 94%, *p* = 0.15; PDC > 80% 81% vs. 87%; *p* = 0.5) at 12 months from the index encounter (Table 2).

### Patient interviews

We summarized findings from the qualitative patient interviews in two themes that portray patient perspectives on decision making. They are described below using the lens of patient competencies, focusing on knowledge, skills or abilities that patients need to engage in SDM [24]. The competencies reflected most clearly in our data include the ability to systematically articulate the problem and expectations, and the ability to negotiate decisions.

#### Articulating the problem and expectations in a systematic manner

Patients in both study arms described communicating their health concerns to clinicians, including symptoms of high blood sugar levels, and they gave examples of “good” doctors who explained medications to them and answered any questions they had. In the intervention arm, patients provided more and more specific examples of information sharing during the encounter, compared to more general comments about issues like side effects in the usual care arm.

The following two patients demonstrate some of the identified differences in intervention and usual care arm conversations. Patient 14 talked about getting support from his clinician and his daughters, but ultimately, he felt he just needed to better follow through with his clinician’s advice to lose weight. His quote (Table 3, 1a) exemplifies the fact that he usually knows how the clinical conversation is going to play out. Patient 6 provided a contrasting example of sharing information in the encounter (Table 3, 1b). She spoke about how the conversation aid helped her talk with her clinician about medication costs. In fact, she thought the tool provided education for both of them.

**Table 3:** Selected quotes from patient interviews

	**Articulating the problem and expectations in a systematic manner**
1a	“Sometimes, I tell the doctor I think I am taking too many. He just looks at me and ‘Mhmm. Exercise, exercise’”. (Patient 14 (male), usual care)
1b	“I know that we talked about the cost of some of these. you know she mentioned the fact that this is a really good med, but it’s also $9 a day to use. So you know, right now I’m using Metformin and it’s seems to be doing the job. At a dime a day so, what do you do? [The conversation with the SDM tool] was a little bit different because we kind of used these cards and there was some kind of a little survey about what was the most important… I don’t think she was aware of how expensive meds are. Um, she just prescribed a cream for like a heat rash. And it was a little 4 ounce tube, it was $40. I asked her before the conversation ‘How much do you think that costs?’ and she said ‘Oh, probably $5’ and it was $40 so, I don’t know that doctors realize how expensive this stuff is”. (Patient 6 (female), intervention)
	**Negotiating decisions**
2a	“So, my levels were kind of high and at that time he had talked about increasing my level of medications. And so I told him I needed….he gave me 3 months to try to get it back under control. Which I did….You know, I’m the one that is in control, because I know what I need to do. I just need to do it”. (Patient 16 (male), usual care)
2b	“Oh, yah…yah. She knows that I will. [Laughs] I am very blunt. Blunt about what I want and what I don’t want. She knows. She’s very—she is also very blunt in telling me what I need. We kind of go back and forth.” […] Well, she just kind of made that decision. And, she said that she wanted me to try the Glyburide because I have been refusing to go on insulin, because I don’t want to go on insulin…so she told me—I think she gave…she said, like, a couple different medications and then she decided on the Glyburide”. (Patient 13 (female), usual care)
2c	“Well, the three things I picked out was cause, side effects, and I can’t remember the third one I picked out. But then she recommended that one medicine and I said, yah, I’d consider taking that one because it was a tablet form; it wasn’t injection. And it could be added to—in addition to the medicine I take now. It would be another tablet—another medicine… and there wasn’t many side effects that I was concerned about where other medicine you gain weight, you have dizziness and nauseating and I didn’t want any of that”. (Patient 2 (female), intervention)

#### Negotiating decisions

Patients in both study arms described negotiating decisions in the clinical encounter, especially making an argument when they wanted to try a strategy a little longer before increasing a medication dose or going to an injectable. They often placed these discussions in the context of on-going negotiations with the clinician on these topics. Patient 16 described a 3-month window that he had previously requested, which at this visit was extended another 6 months (Table 3, 2a). Patient 13 described the open communication she has with her clinician, including discussion of her preferences (i.e., refusal to go on insulin). Still, when it came to the medication decision, she largely left it to her clinician (Table 3, 2b).

Patients in the intervention arm described similar competencies in negotiating disease management decisions but they also described the role that the conversation aid had when focusing the conversation on the most important decision-making factors. Patient 2 referenced prior negotiations (“*I had told her that I did not want to take insulin injections. She knew that from previous doctor appointments*.”) but then went on to describe how her clinician used the conversation aid to talk about other options (Table 3, 2c). Patient 1 described her use of the conversation aid outside of the encounter. She reviewed the side effects information on the tool with her clinician and decided not to make a change. A few days later, though, she reviewed it again and then called her clinician to say she was ready to make a change.

## Discussion

### Main findings

We found that the use of the Diabetes Medication Choice SDM conversation aid by patients with type 2 diabetes and their clinicians during primary care encounters promoted patient knowledge and clinicians’ effort to involve patients in decision making, without adding to the length of the encounter. However, the use of the conversation aid had no significant effect on satisfaction, treatment selection, adherence to treatment, or glycemic control. Qualitative analyses highlighted differences in the way clinicians involved patients in decisional conversations, and patients using the conversation aid were able to articulate what they learned about different medications as their clinician led them through a conversation focused on factors important to them.

### Strengths and limitations

This study has some limitations. We did not reach our targeted sample size and there was substantial missing data resulting in loss of precision. Funding affected the completion of the trial as planned and the timely reporting of its findings. Despite enrolling a more diverse population, as in our previous trials, participants had high adherence at baseline and limited room to improve their glycemic control. And yet, the mean HbA1c in both arms at study end was >8% and fewer than a third of participants achieved HbA1c < 7.3%. Patients and analysts were not blinded to the intervention which may have biased survey responses and analysis of video recordings, including scoring of the OPTION12. Strengths of this trial included its pragmatic randomized design, implementation of allocation concealment, implementation of the intervention in usual primary care settings, analyses accounting for clustering, and magnitude of results comparable to previous efficacy trials.

### Implication for practice, policy, and research

Our findings leave our trial question open. In the time that has elapsed since the conclusion of this trial, important developments have changed the landscape of diabetes care, including the emergence of new agents which differ from the ones available at the time of trial in their cost, administration routes, and side effects including ability to cause hypoglycemia and weight gain. These options reduce the risk of adverse cardiovascular and renal events, outcomes that patients find important [25]. Furthermore, regional guidelines and quality measures have been liberalized to account for the evidence about the impact of glycemic control in patients with type 2 diabetes and the harms associated with overtreatment [26]. Remote care approaches such as telemedicine have become mainstream with the COVID-19 pandemic, and yet little is known about the feasibility and effectiveness of remote SDM approaches [27]. That there are more ways of caring and that the tools used in this study require little clinician training and no patient preparation may all contribute to promote SDM in diabetes care even where this expectation may be just evolving [28]. Also, it has become clear that adherence as an outcome measure is, like for other outcomes, quite sensitive to the prevalence of nonadherence at baseline [29]. At the same time, conducting a trial in patients with poor treatment fidelity at baseline poses substantial challenges. For example, patients’ decision to participate in a trial, and their fidelity to participation test some of the same processes that contribute to treatment adherence. New minimally disruptive research methods may need to be developed and implemented to better test interventions seeking to improve the care of patients struggling to implement treatment work with limited capacity to do so [30].

Finally, the care of patients with diabetes is evolving towards “whole-person care” in response to the increased recognition of their substantial personal and biological complexity [31]. As patients struggle with multimorbidity, social isolation, and treatment burden, it is important to build treatment programs that can be weaved into the challenging lives of these patients. Thus, SDM becomes less about choosing a drug and more about co-crafting a treatment program so that it can solve the problems the patient faces in a manner that makes intellectual, emotional, and practical sense to that patient [32]. Our team has proposed the notion that in these patients, SDM is not about making choices but about addressing the problematic situation of patients [33]. In this way, SDM joins other approaches clinicians can use, including minimally disruptive medicine [34], to improve the effectiveness of diabetes care by making care fit. Subsequent efforts to assess the value of SDM in the care of patients with diabetes may therefore need to select patients for whom usual care approaches (target driven, mechanistically or algorithmically directed) fail (e.g., patients with poor glycemic control, low treatment satisfaction, low treatment fidelity) and test the value of “problem-solving” SDM [33]. This manner of care would involve a toolkit of approaches that work together to help the patient and clinician in defining which aspect of the patient’s situation requires action and which action that situation requires. Outcomes may need to include not just glycemic control parameters, which are insensitive to the benefits of specific diabetes agents, but also the human quality of care and the degree of fit the treatment exhibits in patients’ lives.

## Conclusions

An implementation in primary care of an SDM conversation tool about diabetes medications was able to improve clinician involvement of patients in diabetes care decisions and to improve patient knowledge without impacting visit duration but this did not translate into changed treatments or improved diabetes control or treatment adherence in patients with type 2 diabetes. The effectiveness question our trial asked remains open. Our results strongly suggest methodological modifications to make subsequent trials more successful, for example by focusing the intervention among patients who exhibit signs of poor treatment fit, including low satisfaction with treatment, high treatment burden, poor treatment fidelity, and poor disease control.

## Data Availability

The data that support the findings of this study are available from the corresponding author, NDS, upon reasonable request.

## Appendix A. Patient interview guide

*Note that the goal of an open-ended “qualitative” interview guide is to promote discussion around a pre-determined set of content domains or topics [marked in brackets]. The questions will be tailored to each participant and thus the scripts presented here may be modified depending on the response of the interviewee. Each domain is covered in the conversation between research participant and interviewer. Probes (clarification questions) are used to make certain that the interviewer understands the answers offered. Not every probe will be asked. This style of interviewing is used when studying a new area of inquiry where little previous research has been done. One goal is to elicit concerns that were not anticipated by the researchers*.

*N.B. The time allocated to each interview is ~30* *min. The length of the interview can vary depending on the interviewee. This interview guide is intended to provide a road map for the interviewer, but not all questions will be asked in all cases, nor will all probes be used*.

*If the patient’s enrollment visit was videotaped, the interviewer may ask the patient to view a vignette of the patient–clinician encounter*. “At your last visit, you agreed to have it videotaped. I’d like to play a short part of the video where your provider is showing you the diabetes cards and discussing care options. We think this will help remember the discussion. At any time we can turn the video off”.

**Introductions**

Thank you for participating in the Diabetes study.

My name is xxx. I am a [medical anthropologist and] qualitative researcher for Mayo Clinic. I will ask you a few questions about your recent visit with your doctor. Is that okay?

In our study, we look at different ways to share information about diabetes

medications so that we can improve the material. We are especially interested what patients and doctors do when they decide about choosing diabetes medications.

**General questions**

But, before I will be asking you a few questions about your visit with your doctor, I have a few general points to go through to make sure you are okay with the interview.

Do you have any questions about the study or the interview?

About the procedures we plan to use to protect your confidentiality?

Do I have your permission to record our conversation?

If at any moment you would like to stop the tape, please let me know. We can always stop it and turn it back on. Okay?

Thanks. [turn on tape]

**Okay, let’s get started**.

(1) [motivation] Have you been in research studies before? Why did you decide to participate in this one?

(2) What have you heard about the idea of using educational handouts or other materials to make decisions about choosing medications?

(3) (USUAL CARE) Did your doctor give you educational/informational handouts or write down information for you during your last visit?

Probe: What about showing you something on the computer?

(3) (INTERVENTION) Did your doctor use the diabetes medication choice cards with you at your last visit? Had you seen something like this before?

**Now, before we talk more about the cards and the visit with your doctor, let’s talk a bit about how you like to take care of your health:**

(4) [Action] So first, can you tell me about your history of diabetes (INSERT YEARS)? How did it start?

Probe: How do you cope with your diabetes on a daily basis?

(5) What type of work do you do? Does that make it difficult for you to manage your diabetes (med intake, food intake, exercise?)

(6) Do you live alone? [AGE?].

Probe: Is someone helping you with your food prep/ medications?

(7) How many medications total do you take? Daily? [from video, patient takes XX different medications and/or a daily aspirin]

Probes: Do you also have a history of high blood pressure? Do you have a history of high cholesterol?

**We will now turn to some questions about health decision-making:**

(8) [Decision-making preference] What do you usually do to help you make decisions about your health? Can you give an example?

For example:

Do you read up online?

Do you talk to friends and family about it?

Do you talk to your doctor/health professional?

(8a) If you are unsure about what to do, how do you ordinarily make a decision about your health? Example?

(9) Can you describe a typical medication discussion with your doctor? Who, in your view, should be in charge?

(9a) When you think of your last visit, was the medication discussion from your usual medication discussion with your doctor?

(10) Do you feel that you can speak your mind when discussing medication options with your doctor? Can you give an example?

Probe: When discussing new medication options, do you feel pressured into making the decision your doctor recommends?

(11) [Participation in decision-making] How much guidance or independence do you feel you need when making an important decision about your health? Can you give an example?

For example:

Do you like to discuss things with your doctor?

Do you want your doctor to tell you what to do?

Do you prefer to decide on your own?

(11b) [social network] Who would you say are the three most important people involved in helping you to manage your diabetes and stay healthy?

Probe: Explain. Why do you consider these three so important?

**(USUAL CARE) Now let’s talk a bit more about your last visit with your doctor:**

(12) [Action] You came to see your clinician about INSERT DATE ago to do a checkup, blood tests etc. Can you describe the visit in your own words?

(13) [sense-making] Can you tell me what you and your doctor talked about? [*adding a new med, increasing dose, side effects, risks and benefits]?*

(14) [sense-making] IF APPLICABLE ONLY

Did you discuss the possibility of XXX [ADJUST I.E. adding a new medication]. What do you remember about the different medications, their pros and cons?

Probes: Do you remember what s/he said?

Did s/he write anything down?

Did s/he give you options?

(15) Did you participate in the decision to the extent that you wanted? How so?

(15a) [if applicable[Which medication did you add/change? (INSERT MEDICATION NAME)

Probes:

Are you on it now? How do you feel about it?

Do you regret your decision to having added this particular medication? Explain.

(16) [sense-making] What in the medication discussion helped you to decide whether you should take a different diabetes medication?

(17) Do you remember any specific issues about medications that were discussed?

Probe: Weight loss, cost, schedule, etc. (Some medications can affect your weight, did you discuss this? What about cost?)

(18) Were you able to accomplish what you had hoped to do during this visit?

(19) [coherence/interaction] How would you describe your relationship with your clinician?

(20a) Did you feel that you got adequate information from your clinician about diabetes medications and their potential risks and benefits? How so?

(20b) Did you feel that your clinician took enough time to talk to you? Explain. (Visit lasted about XX minutes)

(20) Would you say you have changed anything about how you take care of yourself since you came to see your doctor in DATE?

Probe: If yes, what have you changed?

If no, do you plan to change anything about how you take care of yourself? What?

(21) [social network] Do you talk about your diabetes medications with your friends and family?

Probes: Why? Why not?

What do you talk about?

(22) [appraisal] Is there any way you regret how your last clinic visit went? Explain.

Probe: Would you have wished the visit would have gone differently? Explain.

(23) Is there anything you would like to add? Any topics, you feel, I should have addressed?

Thank you for your time. You can now choose between these two cookbooks as a little gift for your participation in this study…

**Follow-up:** We plan to call you again in a few months for a follow-up over the phone. Is that okay?

Please feel free to contact the Mayo Clinic study coordinator or myself if you have any questions about this study. My Mayo phone: 507-XXX-XXXX

**(INTERVENTION) Now let’s talk a bit more about your last visit with your doctor:**

(12) [Action] You came to see your clinician about INSERT DATE ago to do a checkup, blood tests etc. Can you describe the visit in your own words?

Probe: What did you talk about?

What were issues that came up?

(13) [sense-making] Our records indicate that you used a diabetes decision making tool at your clinic visit. What do you remember about it [*show patient tool. Let patient look at and play with it while talking*]?

Probes: Can you tell me what you and your doctor talked about when looking at the tool [*side effects, risks and benefits*]?

Do you remember what s/he said?

Did s/he write on it?

Did s/he hand this to you?

(14) [sense-making] So when you looked at this tool, did it make sense to you?

Probes: Was it easy/hard to understand? Explain.

(15) [sense-making] IF APPLICABLE ONLY

Did you discuss the possibility of XXX [ADJUST I.E. adding a new medication]. What do you remember about the different medications, their pros and cons?

Probes: Did you participate in the decision to the extent that you wanted? How so?

(15a) [if applicable[Which medication did you add/change? (INSERT MEDICATION NAME)

Probes:

Are you on it now? How do you feel about it?

Do you regret your decision to having added this particular medication? Explain.

(16) [sense-making] Would you say that the tool helped you to decide whether you should take a different diabetes medication?

Probes: In your view, are there downsides of taking this diabetes medication?

What are the positive sides of taking this diabetes medication?

(17) Did you decide to take a new diabetes medication?

Probe: Why? Why not?

Did you participate in the decision to the extent that you wanted?

(18) Medications can be quite expensive. Did you look at the cost card?

Probes: How do you usually discuss the cost of your medications with your clinician?

(19) [Action] Diabetes medications can influence your weight loss or gain. Did you look at the weight card?

Probe: How do you usually discuss weight issues with your clinician?

**(INTERVENTION) We also hope to understand how this tool may influence your clinic appointment**.

(20) [interaction] Looking back, do you think that using the diabetes cards changed your clinic visit in any way? If so, how?

Probes: Were you able to accomplish what you had hoped to do during this visit?

(21) [coherence/interaction] How would you describe your relationship with your clinician?

Probe: Would you say that using the diabetes cards affected your relationship with your clinican? If so, how? Explain.

(21a) Did you feel that you got adequate information from your clinician about diabetes medications and their potential risks and benefits? How so?

(21b) Did you feel that your clinician took more/less time to talk to you? Explain. (Visit lasted about XX minutes)

(22) Would you say you have changed anything about how you take care of yourself since you came to see your doctor in DATE?

Probe: If yes, what have you changed?

If no, do you plan to change anything about how you take care of yourself? What?

(23) [social network] Would you recommend using the diabetes cards to your friends and family?

Probes: Why? Why not?

What would you tell them about the cards?

Your clinician gave you a brochure. Did you show them the brochure your clinician gave you?

(24) [appraisal] Is there any way you regret using the diabetes cards during your clinic visit? Explain.

Probe: Would you have wished the visit would have gone differently? Explain.

(25) Is there anything you would like to add? Any topics, you feel, I should have addressed?

Thank you for your time. You can now choose between these two cookbooks as a little gift for your participation in this study…

**Follow-up:** We plan to call you again in a few months for a follow-up over the phone. Is that okay?

Please feel free to contact the Mayo Clinic study coordinator or myself if you have any questions about this study. My Mayo phone: 507-XXX-XXXX

## Appendix B. 2- and 6-month-patient surveys

Tables 4, 5

**Table 4:** Two-month-survey responses

	Usual care (*N* = 161)	Conversation aid (*N* = 189)	Total (*N* = 350)
At the time you enrolled in the study, you decided to start taking a diabetes medicine, are you still taking this medicine?			
Missing	117 (72.7%)	147 (77.8%)	264 (75.4%)
Yes	34 (21.1%)	36 (19.0%)	70 (20.0%)
No	10 (6.2%)	6 (3.2%)	16 (4.6%)
At the time you enrolled in the study, you decided not to begin a new diabetes medicine. Are you taking a new medicine at this time?			
Missing	95 (59.0%)	126 (66.7%)	221 (63.1%)
Yes	12 (7.5%)	15 (7.9%)	27 (7.7%)
No	54 (33.5%)	48 (25.4%)	102 (29.1%)
What was the main reason for stopping this medicine?			
Missing	148 (91.9%)	177 (93.7%)	325 (92.9%)
Cost	0 (0.0%)	2 (1.1%)	2 (0.6%)
Side effects	5 (3.1%)	2 (1.1%)	7 (2.0%)
Feel fine	0 (0.0%)	1 (0.5%)	1 (0.3%)
Trouble filling Rx	1 (0.6%)	1 (0.5%)	2 (0.6%)
Can’t remember to take	2 (1.2%)	1 (0.5%)	3 (0.9%)
Other reason	5 (3.1%)	5 (2.6%)	10 (2.9%)
If you are taking a diabetes medication, how many medications do you think you missed within the last week?			
Missing	60 (37.3%)	94 (49.7%)	154 (44.0%)
0	76 (47.2%)	69 (36.5%)	145 (41.4%)
1	10 (6.2%)	11 (5.8%)	21 (6.0%)
1.5	0 (0.0%)	1 (0.5%)	1 (0.3%)
2	7 (4.3%)	8 (4.2%)	15 (4.3%)
3	7 (4.3%)	6 (3.2%)	13 (3.7%)
6	1 (0.6%)	0 (0.0%)	1 (0.3%)
How would you rate your health?			
Missing	46 (28.6%)	84 (44.4%)	130 (37.1%)
Excellent	3 (1.9%)	2 (1.1%)	5 (1.4%)
Very good	24 (14.9%)	25 (13.2%)	49 (14.0%)
Good	58 (36.0%)	49 (25.9%)	107 (30.6%)
3.5	1 (0.6%)	0 (0.0%)	1 (0.3%)
Fair	28 (17.4%)	22 (11.6%)	50 (14.3%)
Poor	1 (0.6%)	7 (3.7%)	8 (2.3%)

**Table 5:** Six-month survey responses

	Usual care (*N* = 161)	Conversation aid (*N* = 189)	Total (*N* = 350)
At the time you enrolled in the study, you decided to start taking a diabetes medicine, are you still taking this medicine?			
Missing	126 (78.3%)	154 (81.5%)	280 (80.0%)
Yes	25 (15.5%)	27 (14.3%)	52 (14.9%)
No	10 (6.2%)	8 (4.2%)	18 (5.1%)
At the time you enrolled in the study, you decided not to begin a new diabetes medicine. Are you taking a new diabetes medicine at this time?			
Missing	93 (57.8%)	126 (66.7%)	219 (62.6%)
Yes	27 (16.8%)	18 (9.5%)	45 (12.9%)
No	41 (25.5%)	45 (23.8%)	86 (24.6%)
If stopped, what was the main reason for stopping this medicine?			
Missing	149 (92.5%)	178 (94.2%)	327 (93.4%)
Cost	1 (0.6%)	2 (1.1%)	3 (0.9%)
Side effects	4 (2.5%)	3 (1.6%)	7 (2.0%)
Can’t remember to take	1 (0.6%)	1 (0.5%)	2 (0.6%)
Other reason	6 (3.7%)	5 (2.6%)	11 (3.1%)
How many diabetes medicines do you think you missed within the last week?			
Missing	66 (41.0%)	92 (48.7%)	158 (45.1%)
0	73 (45.3%)	71 (37.6%)	144 (41.1%)
1	11 (6.8%)	13 (6.9%)	24 (6.9%)
2	8 (5.0%)	5 (2.6%)	13 (3.7%)
≥3	3 (1.8%)	8 (4.1%)	11 (3.3%)
How would you rate your health?			
Missing	54 (33.5%)	88 (46.6%)	142 (40.6%)
Excellent	1 (0.6%)	3 (1.6%)	4 (1.1%)
Very good	24 (14.9%)	25 (13.2%)	49 (14.0%)
Good	59 (36.6%)	45 (23.8%)	104 (29.7%)
Fair	23 (14.3%)	24 (12.7%)	47 (13.4%)
Poor	0 (0.0%)	4 (2.1%)	4 (1.1%)
Have you used these cards with your clinician?			
Missing	109 (67.7%)	127 (67.2%)	236 (67.4%)
Yes, one time	1 (0.6%)	8 (4.2%)	9 (2.6%)
Yes, more than one time	2 (1.2%)	4 (2.1%)	6 (1.7%)
No	49 (30.4%)	50 (26.5%)	99 (28.3%)
Knowledge (106/103), % correct	50.3 (46.3, 54.3)	55.2 (51.1, 59.2)	52.7 (49.9, 55.6)
