# Correction to: Making sense of diabetes medication decisions: a mixed methods cluster randomized trial using a conversation aid intervention

**DOI:** 10.1007/s12020-022-03240-3

**Published:** 2022-11-10

**Authors:** Marleen Kunneman, Megan E. Branda, Jennifer L. Ridgeway, Kristina Tiedje, Carl R. May, Mark Linzer, Jonathan Inselman, Angela L. H. Buffington, Jordan Coffey, Deborah Boehm, James Deming, Sara Dick, Holly van Houten, Annie LeBlanc, Juliette Liesinger, Janet Lima, Joanne Nordeen, Laurie Pencille, Sara Poplau, Steven Reed, Anna Vannelli, Kathleen J. Yost, Jeanette Y. Ziegenfuss, Steven A. Smith, Victor M. Montori, Nilay D. Shah

**Affiliations:** 1 Knowledge and Evaluation Research Unit, Division of Endocrinology, Diabetes, Metabolism, and Nutrition, Mayo Clinic, Rochester, MN USA; 2 Medical Decision Making, Department of Biomedical Data Sciences, Leiden University Medical Center, Leiden, The Netherlands; 3 Department of Biostatistics and Informatics, Colorado School of Public Health, University of Colorado-Denver Anschutz Medical Campus, Aurora, CO USA; 4 Division of Biomedical Statistics and Informatics, Department of Health Sciences Research, Mayo Clinic, Rochester, MN USA; 5 Division of Health Care Policy and Research, Department of Health Sciences Research, Mayo Clinic, Rochester, MN USA; 6 Laboratoire d’anthropologie des enjeux contemporains, Lyon, France; 7 Faculty of Public Health and Policy, London School of Hygiene and Tropical Medicine, London, UK; 8 Department of Medicine, Hennepin Healthcare and University of Minnesota, Minneapolis, MN USA; 9 Mayo Clinic Robert D. and Patricia E. Kern Center for the Science of Health Care Delivery, Rochester, MN USA; 10 Department of Psychiatry and Psychology, Mayo Clinic Health System, Mankato, MN USA; 11 Department of Family Medicine and Community Health, University of Minnesota Medical School, Minneapolis, MN USA; 12 Practice-Based Research Network, Mayo Clinic, Rochester, MN USA; 13 Center for Translational Science Activities, Mayo Clinic, Rochester, MN USA; 14 Center for Patient and Provider Experience, Hennepin County Medical Center, Minneapolis, MN USA; 15 School of Nursing, University of Minnesota, Minneapolis, MN USA; 16 Decision Partners for Health, Richfield, MN USA; 17 Mayo Clinic Health System Northwest Wisconsin, (dept) Home Health and Hospice, Eau Claire, WI USA; 18 Department of Family and Emergency Medicine, Faculty of Medicine, Laval University, Quebec, QC Canada; 19 Park Nicollet International Diabetes Center, St. Louis Park, MN USA; 20 Mayo Clinic Health System, La Crosse, WI USA; 21 Kern Center for the Science of Health Care Deliver, Mayo Clinic, Rochester, MN USA; 22 Office of Professional Worklife, Hennepin Healthcare, Minneapolis, MN USA; 23 Department of Internal Medicine, Park Nicollet Clinic, Brooklyn Center, MN USA; 24 Division of Epidemiology, Department of Health Sciences Research, Mayo Clinic, Rochester, MN USA; 25 Division of Health Sciences Research, Mayo Clinic, Rochester, MN USA; 26 Center for Evaluation and Survey Research, HealthPartners Institute, Bloomington, IN USA; 27 Division of Endocrinology, Diabetes, Metabolism, and Nutrition, Mayo Clinic, Rochester, MN USA

Correction to: Endocrine 2022 75(2):377–391 10.1007/s12020-021-02861-4

After publication of this article, it came to the authors’ attention that they had included the incorrect Clinical Trial Registration number in the original article.

The correct ClinicalTrials.gov number is: NCT01293578

